# Associations between smoking behavior-related alleles and the risk of melanoma

**DOI:** 10.18632/oncotarget.10144

**Published:** 2016-06-17

**Authors:** Wenting Wu, Hongliang Liu, Fengju Song, Li-Shiun Chen, Peter Kraft, Qingyi Wei, Jiali Han

**Affiliations:** ^1^ Department of Epidemiology, Richard M. Fairbanks School of Public Health, Melvin and Bren Simon Cancer Center, Indiana University, Indianapolis, Indiana, USA; ^2^ Duke Cancer Institute, Duke School of Medicine, Durham, North Carolina, USA; ^3^ Department of Epidemiology and Biostatistics, Key Laboratory of Cancer Prevention and Therapy, National Clinical Research Centre of Cancer, Tianjin Medical University Cancer Institute and Hospital, Tianjin, P. R. China; ^4^ Department of Psychiatry, Washington University School of Medicine, St. Louis, Missouri, USA; ^5^ Department of Epidemiology, Harvard School of Public Health, Boston, Massachusetts, USA; ^6^ Channing Division of Network Medicine, Department of Medicine, Brigham and Women's Hospital, Boston, Massachusetts, USA; ^7^ Department of Biostatistics, Harvard University School of Public Health, Boston, Massachusetts, USA

**Keywords:** risk of melanoma, smoking behavior, single-nucleotide polymorphisms (SNPs), case-control study, CHRNA5-A3-B4 gene cluster

## Abstract

Several studies have reported that cigarette smoking is inversely associated with the risk of melanoma. This study further tested whether incorporating genetic factors will provide another level of evaluation of mechanisms underlying the association between smoking and risk of melanoma. We investigated the association between SNPs selected from genome-wide association studies (GWAS) on smoking behaviors and risk of melanoma using 2,298 melanoma cases and 6,654 controls. Among 16 SNPs, three (rs16969968 [A], rs1051730 [A] and rs2036534 [C] in the 15q25.1 region) reached significance for association with melanoma risk in men (0.01 < = *P* values < = 0.02; 0.85 < = Odds Ratios (ORs) <= 1.20). There was association between the genetic scores based on the number of smoking behavior-risk alleles and melanoma risk with *P*-*trend* = 0.005 among HPFS. Further association with smoking behaviors indicating those three SNPs (rs16969968 [A], rs1051730 [A] and rs2036534 [C]) significantly associated with number of cigarettes smoked per day, CPD, with *P* = 0.009, 0.011 and 0.001 respectively. The SNPs rs215605 in the *PDE1C* gene and rs6265 in the *BDNF* gene significantly interacted with smoking status on melanoma risk (interaction *P* = 0.005 and *P* = 0.003 respectively). Our study suggests that smoking behavior-related SNPs are likely to play a role in melanoma development and the potential public health importance of polymorphisms in the *CHRNA5-A3-B4* gene cluster. Further larger studies are warranted to validate the findings.

## INTRODUCTION

Melanoma is the most lethal form of skin cancer. Incidence and mortality due to melanoma are rising recently in whites [1]. The major host risk factors for melanoma include pigmentation phenotypes, number of melanocytic nevi and a family history of melanoma [2–5]. Tobacco smoke contains numerous carcinogenic compounds and is one of the most important factors for multiple cancers and many other severe diseases [6, 7]. Though smoking is associated with an elevated risk of most cancers, some epidemiological studies have reported a decreased risk of cutaneous malignant melanoma (MM) among smokers [8–11]. Our previous finding showed an inverse association between smoking and melanoma risk, especially on the body site of head and neck [10].

Meanwhile recently, consistent evidence has suggested a genetic basis for smoking behaviors. A few studies have reported associations between polymorphisms in the *CHRNA5-A3-B4* gene cluster and serum cotinine levels, smoking intensity, suggesting a genetic basis for smoking behaviors [7, 12, 13]. Genetic polymorphisms in the *CHRNA5* is associated with intensity of smoking and nicotine dependence. The strongest associations previously published are for rs16969968 and rs1051730 with smoking quantity [7, 14]. The rs6265, a nonsynonymous SNP in the *BDNF* gene, was strongly associated with smoking initiation, and rs7872903 near the *DBH* gene was significantly associated with smoking cessation [15]. Incorporating genetic factors will provide another level of evaluation of mechanisms underlying the association between smoking and melanoma risk. Hence, we performed an association study on smoking behavior-related genetic variants selected from recent genome-wide association studies (GWAS) and the risk of melanoma. Furthermore, five harmonized smoking phenotypes: smoking initiation, number of cigarettes smoked per day (CPD) and pack years, years since quit smoking in former smokers and smoking cessation were investigated in our study. We analyzed associations between those SNPs and each smoking phenotype, as well as SNP–by–smoking interaction to comprehensively understand the relations between SNPs, smoking behaviors and risk of melanoma.

## RESULTS

### Population characteristics

We combined data from two GWAS on melanoma: the nested case-control study within the Nurses’ Health Study (NHS) and Health Professionals Follow-up Study (HPFS) (494 cases and 5,628 controls) and the MD Anderson Cancer Center melanoma case-control study (1,804 cases and 1,026 controls) (Table 1, Supplementary Material: Table S1) [16]. Detailed descriptions of these two studies are presented in the online Supplementary Methods (Supplementary Material). Single-nucleotide polymorphisms (SNP) significantly associated with smoking phenotype in Caucasian populations were selected from five existing GWAS and meta-GWAS.

**Table 1:** Characteristics of melanoma cases and controls in the NHS and HPFS

	NHS and HPFS		
	All *N* = 6122	Cases *N* = 494	Controls *N* = 5628
Male (*n*,%)	2428 (39.66)	177 (35.83)	2251 (40.00)
Female (*n*,%)	3694 (60.34)	317 (64.17)	3377 (60.00)
Age (mean ± SD)	43.46 ± 7.69	43.12 ± 7.86	43.49 ± 7.67
Family history of melanoma (%)	7.53	17.2	6.68
**Smoking Variables**			
Smoking status			
Never (*n*,%)	2628 (47.17)	224 (47.36)	2404 (47.16)
Past smoker (*n*,%)	2351 (42.20)	214 (45.24)	2137 (41.92)
Current smoker (*n*,%)	592 (10.63)	35 (7.40)	557 (10.93)
Ever Smoker (*n*,%)	2943 (52.83)	249 (52.64)	2694 (52.85)
Duration of Smoking			
Never (*n*,%)	2628 (48.19)	224 (48.07)	2404 (48.21)
< 20 years (*n*,%)	1316 (24.13)	116 (24.89)	1200 (24.06)
20–29 years (*n*,%)	536 (9.83)	52 (11.16)	484 (9.70)
30+ years (*n*,%)	973 (17.84)	74 (15.88)	899 (18.03)
Pack years for smokers (Mean ± SD)	16.91 ± 19.25	15.47 ± 17.86	17.05 ± 19.38
Cigarettes Smoked Per day in ever smokers			
Never (*n*,%)	2628 (47.17)	224 (47.16)	2404 (47.36)
1–14 (*n*,%)	1282 (23.01)	119 (22.81)	1163 (25.16)
15 + (*n*,%)	1661 (29.82)	130 (30.03)	1531 (27.48)
Yeas since quitting in past smokers			
10 + years (*n*,%)	1773 (75.74)	156 (72.90)	1617 (76.02)
< 10 years (*n*,%)	568 (24.26)	58 (27.10)	510 (23.98)

### Association of selected loci with risk of melanoma

Power calculations showed that our combined samples had an 80% power to detect variants, conferring an OR of 1.18 with an allele frequency of 10%. SNPs rs16969968 and rs1051730 in the 15q25.1 *CHRNA5-A3-B4* gene cluster reached nominal significance (OR = 0.91, 95% CI (0.83–0.99), *P* = 0.03 and OR = 0.91, 95% CI (0.84–1.00), *P* = 0.05). Rs2036534, another SNP located in 15q25.1, was also associated with an increased risk of melanoma, OR = 1.14 (95% CI: 1.03–1.26; *P* = 0.01) (Table 2). When we stratified by sex, we observed stronger association between SNPs rs1051730, rs578776 and rs6495308 in the *CHRNA3* gene, rs16969968 in the *CHRNA5* gene, rs2036534 in *HYKK* gene and risk of melanoma among men in the HPFS (OR for rs1051730 = 0.77, 95% CI (0.60–0.97) (*P* = 0.03); OR for rs578776 = 1.44, 95% CI (1.14–1.80) (*P* = 0.002); OR for rs6495308 = 1.44, 95% CI (1.13–1.83) (*P* = 0.003); OR for rs16969968 =0.74, 95% CI (0.58–0.94) (*P* = 0.014); and OR for rs2036534= 1.39, 95% CI (1.09–1.77) (*P* = 0.008). After Bonferroni correction (*P* for statistical significance < 0.05/16 SNPs), a marginally significant association with risk of melanoma was observed for rs6495308 and rs578776 (Tables 2, 3 and Supplementary Material: Table S2). We examined the potential cumulative effects of the 14 independent SNPs in the NHS and HPFS by examining associations between the genetic score and melanoma risk. The genetic scores in our dataset ranged from 1 to 21. Among men in the HPFS, compared with the lowest genetic score quartile (1–8), ORs for the second (9–10), third (11–12), and fourth (13–21) quartiles were 1.23 (95% CI, 0.73 – 2.07), 1.97 (95% CI, 1.22–3.17), and 1.78 (95% CI, 1.11–2.86), respectively (*P-trend* = 0.005).

**Table 2:** Associations between smoking behavior–related SNPs and melanoma risk

SNP	CHR	Gene	Reference Number	All^c^						Men^c^			Women^c^			CytoBand
				Risk	Ref	Allele frequency	OR	95% CI	*P*	OR	95% CI	*P*	OR	95% CI	*P*	
rs215605^a^	7	PDE1C	[30]	G	T	0.38	1.01	0.92–1.10	0.9	0.99	0.88–1.12	0.88	1.01	0.89–1.15	0.85	7p14.3
rs11782673^b^	8	PTK2B	[17]	G	A	0.17	1.09	0.97–1.22	0.14	1.02	0.87–1.19	0.85	1.14	0.98–1.33	0.09	8p21.2
rs6474412^a^	8	SMIM19, CHRNB3	[30]	C	T	0.23	1.02	0.93–1.13	0.63	1.13	0.98–1.3	0.09	0.92	0.8–1.07	0.28	8p11.21
rs13280604^a^	8	CHRNB3	[30]	G	A	0.23	1.02	0.92–1.13	0.69	1.12	0.97–1.28	0.13	0.93	0.8–1.07	0.30	8p11.21
rs7872903^a^	9	FAM163B, DBH	[15]	C	T	0.21	1.00	0.91–1.11	0.98	1.01	0.88–1.17	0.88	0.99	0.86–1.14	0.91	9q34.2
rs1329650^a^	10	HECTD2-AS1	[15]	T	G	0.28	0.97	0.88–1.07	0.54	1.00	0.87–1.14	1.00	0.95	0.83–1.08	0.43	10q23.32
rs1013442^a^	11	BDNF-AS	[15]	T	A	0.25	1.02	0.92–1.13	0.67	1.09	0.95–1.26	0.22	0.97	0.84–1.11	0.66	11p14.1
rs6265^a^	11	BDNF	[15]	T	C	0.19	1.04	0.94–1.16	0.44	1.13	0.97–1.32	0.10	0.97	0.83–1.13	0.67	11p14.1
rs2036534^b^	15	HYKK	[30]	C	T	0.22	**1.14**	**1.03–1.26**	**0.01**	**1.20**	**1.04–1.38**	**0.01**	1.07	0.93–1.24	0.33	15q25.1
rs588765^b^	15	CHRNA5	[14, 17]	T	C	0.42	1.02	0.94–1.11	0.59	1.03	0.91–1.16	0.64	1.00	0.89–1.13	1.00	15q25.1
rs16969968^a^	15	CHRNA5	[15, 17, 30]	A	G	0.35	**0.91**	**0.83–0.99**	**0.03**	**0.85**	**0.75–0.96**	**0.01**	0.99	0.87–1.12	0.86	15q25.1
rs578776^a^	15	CHRNA3	[14]	A	G	0.28	1.08	0.99–1.18	0.1	1.20	0.87–1.66	0.27	1.01	0.89–1.15	0.85	15q25.1
rs1051730^a^	15	CHRNA3	[15, 17, 30]	A	G	0.34	**0.91**	**0.84–1.00**	**0.05**	**0.86**	**0.76–0.98**	**0.02**	0.99	0.88–1.13	0.92	15q25.1
rs6495308^a^	15	CHRNA3	[17]	C	T	0.23	1.08	0.98–1.20	0.11	**1.19**	**1.03–1.36**	**0.01**	0.99	0.86–1.14	0.89	15q25.1
rs7937^a^	19	MIA-RAB4B	[30]	C	T	0.44	0.94	0.86–1.02	0.13	**0.86**	**0.76–0.97**	**0.01**	1.02	0.91–1.15	0.70	19q13.2
rs3733829^a^	19	RAB4B-EGLN2	[15]	G	A	0.36	0.96	0.88–1.04	0.32	0.95	0.84–1.07	0.38	0.96	0.84–1.08	0.47	19q13.2

Abbreviations: CHR, chromosome; OR, odds ratio; SNP, single-nucleotide polymorphism.

a SNPs reached genome-wide signifcance (*P* value = 5 × 10^−8^) with smoking behaviors.

b SNPs did not reach *P* value of 5 × 10^−8^.

c Meta-analysis results for both NHS and HPFS set and MD Anderson set.

**Table 3:** Associations between smoking behavior-related SNPs and melanoma risk in men and women

			Men						Women			
SNP	CHR	Gene	HPFS set				MD Anderson set		NHS set		MD Anderson set	
			Risk	Ref	OR	*P*	OR	*P*	OR	*P*	OR	*P*
rs215605^a^	7	PDE1C	G	T	0.99	0.945	0.99	0.90	0.92	0.36	1.12	0.22
rs11782673^b^	8	PTK2B	G	A	1.04	0.785	1.01	0.96	1.19	0.10	1.09	0.48
rs6474412^a^	8	SMIM19, CHRNB3	C	T	1.36	0.016	1.04	0.69	0.91	0.35	0.94	0.57
rs13280604^a^	8	CHRNB3	G	A	1.34	0.023	1.03	0.76	0.92	0.40	0.94	0.53
rs7872903^a^	9	FAM163B, DBH	C	T	1.08	0.576	0.98	0.86	1.06	0.55	0.93	0.45
rs1329650^a^	10	HECTD2-AS1	T	G	1.09	0.469	0.96	0.63	0.89	0.22	1.01	0.89
rs1013442^a^	11	BDNF-AS	T	A	1.07	0.613	1.10	0.25	0.99	0.89	0.95	0.63
rs6265^a^	11	BDNF	T	C	1.18	0.223	1.11	0.26	0.99	0.89	0.95	0.63
rs2036534^b^	15	HYKK	C	T	**1.39**	**0.008**	1.11	0.23	0.99	0.90	1.18	0.13
rs588765^b^	15	CHRNA5	T	C	0.99	0.959	1.04	0.55	1.03	0.75	0.97	0.74
rs16969968^a^	15	CHRNA5	A	G	**0.74**	**0.014**	0.89	0.14	0.99	0.91	0.99	0.90
rs578776^a^	15	CHRNA3	A	G	**1.44**	**0.002**	1.03	0.71	1.01	0.92	1.02	0.86
rs1051730^a^	15	CHRNA3	A	G	**0.77**	**0.030**	0.90	0.17	1.01	0.90	0.97	0.78
rs6495308^a^	15	CHRNA3	C	T	**1.44**	**0.003**	1.08	0.37	0.96	0.71	1.02	0.85
rs7937^a^	19	MIA-RAB4B	C	T	0.82	0.084	0.88	0.07	0.94	0.44	1.13	0.17
rs3733829^a^	19	RAB4B-EGLN2	G	A	0.96	0.711	0.94	0.41	0.98	0.80	0.93	0.44

a SNPs reached genome-wide signifcance level *(P* value = 5 × 10 ^−8^) with smoking behaviors.

b SNPs did not reach *P* value of 5 × 10^−8^.

### Associations between SNPs and multiple smoking behaviors in the HPFS and NHS

We further analyzed the associations between those SNPs and multiple smoking behaviors in the HPFS and NHS. The SNPs rs1051730, rs16969968 and rs2036534 were significantly associated with smoking quantity (CPD) (*P* = 0.011, *P* = 0.009 and *P* = 0.001 respectively). The SNPs rs6265, rs6495308 and rs7937 were significantly associated with smoking initiation (*P* = 0.017, *P* = 0.032 and *P* = 0.014 respectively). SNP rs2036534 risk allele (C) was associated with smoking cessation (*β* = 0.184, *P* = 0.033), which is consistent with protective effect in smoking quantity (*β* = −0.143, *P* = 0.001). Similar associations was observed for pack years when compare with another smoking quantity phenotype CPD (Table 4).

**Table 4:** Associations between smoking behavior–related SNPs and smoking behaviors among controls of NHS and HPFS

SNP	Risk	Ref	Related Smoking Phenotypes From Literature^a^	CPD^b^^,^^c^			Smoking Cessation^b^^,^^c^			Smoking Initiation^b^^,^^c^			Pack Years^b^^,^^c^			Years since Quit Smoking^b^^,^^c^		
				*β*	s.e.	*P*	*β*	s.e.	*P*	*β*	s.e.	*P*	*β*	s.e.	*P*	*β*	s.e.	*P*
rs215605	G	T	Smoking quantity	−0.002	0.038	0.952	0.113	0.072	0.118	−0.056	0.041	0.176	−0.165	0.536	0.758	0.003	0.027	0.912
rs11782673	G	A	Smoking quantity	−0.036	0.049	0.468	0.064	0.093	0.491	−0.028	0.054	0.596	−0.785	0.698	0.261	0.005	0.035	0.883
rs6474412	C	T	Smoking quantity	**−0.098**	**0.043**	**0.022**	0.072	0.081	0.376	0.039	0.048	0.410	**−1.263**	**0.608**	**0.038**	−0.005	0.030	0.879
rs13280604	G	A	Smoking quantity	**−0.094**	**0.043**	**0.028**	0.073	0.082	0.368	0.041	0.048	0.394	**−1.244**	**0.608**	**0.041**	−0.002	0.030	0.960
rs7872903	C	T	Smoking cessation	0.051	0.044	0.250	−0.110	0.082	0.179	0.030	0.049	0.534	0.921	0.626	0.142	−0.041	0.032	0.196
rs1329650	T	G	Smoking quantity	0.023	0.041	0.571	−0.083	0.076	0.276	0.008	0.045	0.857	0.502	0.579	0.386	0.010	0.029	0.731
rs1013442	T	A	Smoking initiation	**0.084**	**0.042**	**0.044**	−0.130	0.077	0.092	−0.051	0.046	0.265	**1.274**	**0.589**	**0.031**	0.028	0.029	0.341
rs6265	T	C	Smoking initiation	**0.092**	**0.046**	**0.045**	**−0.201**	**0.084**	**0.017**	−0.034	0.050	0.494	**1.723**	**0.646**	**0.008**	0.018	0.033	0.585
rs2036534	C	T	Smoking quantity	**−0.143**	**0.044**	**0.001**	**0.184**	**0.086**	**0.033**	−0.048	0.048	0.318	**−1.649**	**0.626**	**0.009**	−0.024	0.031	0.434
rs588765	T	C	Smoking quantity	−0.021	0.037	0.564	−0.023	0.069	0.735	0.069	0.040	0.086	−0.507	0.518	0.328	−0.025	0.026	0.327
rs16969968	A	G	Smoking quantity	**0.099**	**0.038**	**0.009**	−0.085	0.071	0.234	−0.070	0.042	0.091	**1.699**	**0.536**	**0.002**	0.036	0.027	0.178
rs578776	A	G	Smoking quantity	−0.072	0.040	0.075	0.141	0.078	0.070	−0.029	0.044	0.508	−0.992	0.571	0.083	−0.008	0.028	0.785
rs1051730	A	G	Smoking quantity	**0.097**	**0.038**	**0.011**	−0.107	0.072	0.134	−0.082	0.042	0.051	**1.743**	**0.540**	**0.001**	0.041	0.027	0.134
rs6495308	C	T	Smoking quantity	**−0.100**	**0.043**	**0.021**	**0.181**	**0.084**	**0.032**	0.005	0.047	0.915	**−1.584**	**0.611**	**0.010**	−0.018	0.030	0.557
rs7937	C	T	Smoking quantity	−0.027	0.037	0.467	**−0.170**	**0.069**	**0.014**	0.064	0.040	0.109	0.504	0.522	0.334	0.000	0.026	0.988
rs3733829	G	A	Smoking quantity	−0.071	0.037	0.059	0.050	0.071	0.488	−0.004	0.041	0.928	−0.270	0.531	0.611	−0.018	0.026	0.503

a [14, 15, 17, 30].

b CPD, pack years, and years since quit smoking was analyzed as a continuous variable representing the number of cigarettes smoked per day, number of multiplying years of smoking by the number of cigarettes divided by 20, years since quit smoking in former smokers, respectively. Smoking initiation and smoking cessation were analyzed as dichotomous variables, contrasting ever versus never and former versus current smokers, respectively.

c Analyses were adjusted by age, sex and fve EVs.

### Interaction between SNPs and smoking status in the HPFS and NHS set

We further tested the effect modification by smoking status on the associations between SNPs and risk of melanoma. The SNPs rs215605 in the *PDE1C* gene and rs6265 in the *BDNF* gene significantly interacted with smoking status on melanoma risk (interaction *P* = 0.005 and *P* = 0.003 respectively). After Bonferroni correction, rs6265 was still significant and rs215605 was marginally significant; whereas both SNPs remained significant with FDR correction. Within current smoker, melanoma risk was increased (*β* = 0.694, *P* = 0.006) for rs215605 risk allele G, whereas in never smoker risk the G allele had a weak inverse association with risk (*β* = −0.211, *P* = 0.043). For rs6265 risk allele T, it was associated with an elevated risk of melanoma in never smoker (*β* = 0.275, *P* = 0.019) and had no significant association in both current smokers and ever smokers (Table 5).

**Table 5:** Interaction analysis for smoking status and rs215605 and rs6265

SNP	Risk	Ref	Smoking Status	β (effect size)	SE (standard error)	*P*-value	Sample Size	Interaction *P* value^a^
rs215605	G	T	Never Smoker	**−0.211**	**0.105**	**0.043**	2628	
			Past Smoker	−0.034	0.105	0.745	2351	**0.005**
			Current Smoker	**0.694**	**0.254**	**0.006**	592	
rs6265	T	C	Never Smoker	**0.275**	**0.118**	**0.019**	2628	
			Past Smoker	−0.080	0.135	0.552	2351	**0.003**
			Current Smoker	−0.636	0.375	0.090	592	

a Significant after FDR corrections, FDR *P* = 0.038 for both SNPs.

## DISCUSSION

This study consisted of comprehensive analysis amongst genetic variations, smoking quantity, and risk of melanoma. We observed an inverse association between smoking behavior related genetic variants in 15q25.1 region and risk of melanoma, especially in men, which is consistent with our previous cohort study findings [7, 10].

Cluster of two genes *CHRNA5* and *CHRNA3* on the chromosome 15q25 region, which encodes neuronal nicotinic acetylcholine receptor subunits, is associated with nicotine dependence and smoking quantity [7]. Various meta-analyses based on subjects of European ancestry identified robust associations for rs16969968 in the gene *CHRNA5* and rs1051730 in the *CHRNA3* with smoking quantity (number of cigarettes smoked per day) [7, 14, 15, 17]. Especially, rs16969968, a nonsynonymous variant, was indicated with functions of altering nicotinic receptor conductance *in vitro* by amino acid change in the *CHRNA5* [7, 18, 19]. Those two loci have been found to be associated with lung cancer, chronic obstructive pulmonary disease and body mass index in never smokers [13, 20–23]. Based on our study, both rs1051730 allele (A) in *CHRNA3* gene and rs16969968 allele (A) in *CHRNA5* gene increased smoking quantity and delayed smoking cessation behaviors, while rs2036534 allele (C) in *HYKK* gene reduced smoking quantity and enhanced smoking cessation. Multiple genetic risk factors may be involved in the underlying mechanism for the 15q25.1 region on melanoma development, including increased smoking quantity, and delay in smoking cessation [7, 24, 25].

Meanwhile we detected trends between genetic variations and smoking quantity (smoking intensity and pack years of smoking), smoking cessation and initiation behaviors, to further investigate underlying mechanisms; the results are consistent with previous reports [10, 15, 17]. Inconsistent results were reported previously for *CHRNA5* variants in relation to smoking cessation [7, 26–28]. One previous meta-analysis suggested the rs16969968 allele (A) was correlated with delayed smoking cessation [7]. Our results indicated that both *CHRNA5* variants rs16969968 allele (A) and rs588765 allele (T) were associated with delay in smoking cessation, which validated the previous finding in a large European cohort study. Besides, we found rs2036534 in *HYKK* gene, rs6265 in *BDNF* gene, rs7937 in *MIA-RAB4B* gene and rs6495308 in *CHRNA3*, which previously predicted smoking heaviness or smoking initiation, was also associated with smoking quitting behaviors among ever-smokers. It suggested that multiple genes or genetic variants might mediate smoking cessation [7, 29].

Interestingly, opposite associations of rs215605 in *PDE1C* with risk of melanoma in never and current smokers were observed. An interaction test confirmed that these estimates differed from each other. A previous publication has reported similar phenomena for rs16969968-rs1051730 with an association with body mass index in never smoker [13]. *PDE1C* has been highlighted in a previous GWAS of nicotine dependence and smoking cessation [28, 30]. Meanwhile experimental evidence supports its function in driving cell proliferation, migration and invasion in human malignant melanoma cells, glioblastoma multiforme cells, and etc [31, 32]. Evidence of smoking behavior modification was also identified for rs6265 in *BDNF*. *BDNF* is highly expressed in the brain regions of prefrontal cortex and hippocampus, which is thought to be involved in the cognitive-enhancing effects of nicotine [33]. The rs6265 has been found to be correlated with multiple psychiatric disorders, including schizophrenia, bipolar and eating disorders [34, 35]. Meanwhile, it was recently identified as a genetic risk factor for body mass index [36]. It also indicated that those two SNPs might be weakly associated with risk of melanoma via pathways other than smoking, as well as with changing smoking quantity among smokers, which warrants additional experimental validation.

These results should be interpreted with caution given the multiple testing issues. Meanwhile, the genetic effect of 15q25.1 region was not confirmed in the MD Anderson set, which may be due to heterogeneity of studies. Further large study is warranted to validate these findings. However, to our knowledge, the sample size of this study is among the largest genetic meta-analyses for smoking and melanoma risk investigation. We provided a comprehensive analysis on the relationships between genetic variations, smoking quantity, smoking initiation, smoking cessation and risk of melanoma. Further SNP-smoking interaction analysis suggested a possible modification effect of smoking behaviors.

In conclusion, our study suggested that smoking behavior-related SNPs are likely to play a role in melanoma development. Further studies are warranted to understand the potential functional mechanisms in pathogenesis of melanoma.

## MATERIALS AND METHODS

### Harvard NHS & HPFS sets

#### Description of study populations

##### Nurses’ Health Study

The Nurses’ Health Study (NHS) was established in 1976, when 121 700 female registered nurses between the ages of 30 and 55 years residing in 11 larger US states completed and returned an initial self-administered questionnaire on their medical histories and baseline health-related exposures. Biennial questionnaires with collection of exposure information on risk factors have been collected prospectively. Every 2 years, along with exposures, outcome data with appropriate follow-up of reported disease events are collected. Overall, follow-up has been high; after more than 20 years, ~90% of participants continue to complete questionnaires. From May 1989 through September 1990, we collected blood samples from 32 826 participants in the NHS. Information on melanoma development was first collected in the 1984 questionnaire.

### Health Professionals Follow-up Study

In 1986, 51,529 men from all 50 US states in health professions (dentists, pharmacists, optometrists, osteopath physicians, podiatrists, and veterinarians) aged 40–75 years answered a detailed mailed questionnaire, forming the basis of the study. The average follow-up rate for this cohort over 10 years is > 90%. On each biennial questionnaire, we obtained disease- and health-related information. Between 1993 and 1994, 18,159 study participants provided blood samples by overnight courier. Information on melanoma development was first collected in the 1986 questionnaire. Description of the study population can be found elsewhere [37].

### Melanoma cases and controls in the discovery set

Eligible cases in the NHS and the Health Professionals Follow-up Study (HPFS) consisted of participants with pathologically confirmed invasive melanoma, diagnosed any time after baseline up to the 2008 follow-up cycle for both cohorts. All subjects were United States non-Hispanic Caucasians.

We have previously conducted several GWASs on different disease outcomes (NHS: breast cancer, coronary heart disease, type 2 diabetes, kidney stone, pancreatic cancer, and glaucoma; HPFS: coronary heart disease, type 2 diabetes, kidney stone, advanced prostate cancer, and glaucoma). The study population for eight GWAS sets of the discovery set is presented in following descriptions. We included only controls in each GWAS, except for the kidney stone GWAS, in which we used both cases and controls. Participants without melanoma diagnosis were the controls in the current study, and those with melanoma diagnosis were the cases. In addition, we genotyped the rest of the melanoma cases in both cohorts who were not included in these previous GWASs. The Institutional Review Board at Harvard T.H. Chan School of Public Health and Brigham and Women's Hospital, Harvard Medical School, approved the study protocols.

Informed consent was obtained from all participants. Finally, we included 494 melanoma cases and 5628 controls.

### Laboratory assays

Genotyping in eight GWASs of the discovery set. We performed genotyping in the breast cancer GWAS in NHS using the Illumina HumanHap550 array, as part of the National Cancer Institute's Cancer Genetic Markers of Susceptibility Project. For the coronary heart disease and type 2 diabetes GWASs of the discovery set, we performed genotyping using the Affymetrix 6.0 array. For the glaucoma GWAS, we performed genotyping using the Illumina HumanHap660 array. For the kidney stone, advanced prostate cancer, and melanoma GWASs, we performed genotyping using the Illumina HumanHap610 array. The quality control procedures for eight GWAS sets of the discovery set are presented in following description.

### Imputation and meta-analysis

On the basis of the genotyped single-nucleotide polymorphisms (SNPs) and haplotype information in the National Center for Biotechnology Information build 35 of phase II Hapmap CEU data, we imputed genotypes for > 2.5 million SNPs using the program MACH [38]. Only SNPs with imputation quality *R*^2^ > 0.95 in each study were included in the final analysis. A total of 1 579 307 SNPs were included in the final meta-analysis of the NHS and HPFS.

### M.D. Anderson cancer center set

The study participants for the discovery analysis were from a hospital-based case-control study of melanoma, for which cases were recruited from among non-Hispanic white patients and controls at MD Anderson between March 1998 and August 2008. Samples and data were available from 931 melanoma patients and 1,026 cancer-free controls (friends of other patients reporting to clinics), which were frequency-matched on age and sex, completed a comprehensive skin lifestyle questionnaire, and passed quality control filters for genotyping. This questionnaire was administered by an interviewer to 70% of patients and controls and was self-administered for the remaining 30%. An additional case series comprising 873 individuals presenting for treatment for melanoma at MD Anderson was also included, bringing the total number of melanoma patients to 1,804.

The study protocols were approved by the Institutional Review Board at MD Anderson, and informed consent was obtained from all participants.

Tissue samples were collected as whole blood, with various DNA extraction methods (including Gentra, Qiagen, and phenol/chloroform). DNA samples for the first-stage genome-wide association study were genotyped using the Illumina Omnil-Quad array and were called using the BeadStudio algorithm, at the John Hopkins University Center for Inherited Disease Research (CIDR).

### QC procedures for the M.D. Anderson set

Mean call rate for all samples was 99.86%. Only 41 failed genotyping with > 10% missing rate across all SNPs, and 11 samples had identity problems that could not be resolved. For this study, the IBD coefficients were estimated using 116,002 autosomal SNPs in PLINK (Purcell et al., 2007). In total, 126 duplicated, related (IBD), or outliers identified by PCA were excluded from the study. Following these exclusions there were 1,952 cases and 1,026 controls. Among 2,978 total cases and controls passing quality control, 138 *in situ* cases were subsequently removed from the study for indeterminate phenotype. Ten atypical melanocytic proliferation (AMPs) patients were also excluded as not having invasive cancers. Finally, we analyzed data from 1,804 cases and 1,026 controls available for the association study of melanoma susceptibility (Amos et al., 2011).

### Smoking behavior-related GWASs SNPs selection

A total of 36 SNPs were selected from published GWAS or meta-analysis of GWAS on smoking behaviors [14, 15, 17, 25, 30]. Four carefully harmonized smoking phenotypes were included in this study – smoking initiation (ever versus never been a regular smoker), age of smoking initiation, smoking quantity (number of cigarettes smoked per day, CPD) and smoking cessation (former versus current smokers) – among people of European ancestry. 10 SNPs not available in either melanoma data set were excluded. For SNPs with high linkage disequilibrium (LD), which was characterized by *r*^2^ > 0.8 according to the HapMap 3 (release 2) [39], only one SNP was kept, except for SNPs rs16969968 and rs1051730 located in the CHRNA5-A3-B4 gene cluster. A total of 16 smoking behavior-related SNPs were finally included for analysis.

### Statistical analysis

A total of 16 SNPs were selected and divided into two groups: those associated with smoking phenotype risk with *P* value < 5 × 10^−8^ were in group 1, and those highly associated with smoking phenotype risk that failed to reach that *P* value were in group 2.

For SNPs with a high LD, only those with the smallest *P* value were chosen for the genetic score summary. We used unconditional logistic regression to calculate OR and 95% confidence interval (CI) with adjustment for age, sex, history of melanoma and five eigenvectors (EV), which were calculated for all individuals on the basis of approximately 10,000 unlinked markers with the EIGENSTRAT software. Betas from each study were combined by a meta-analysis with weights proportional to the inverse variance of the beta in each study. We calculated a genetic score on the basis of the presence of total copy number of 14 independent smoking-related risk alleles in the NHS and HPFS. The score was logistically regressed against the melanoma case status, adjusting for age, sex, history of melanoma and five EVs. Subgroup analyses were performed for men and women.

Five carefully harmonized smoking phenotypes were included in further analysis – smoking initiation (ever versus never been a regular smoker), smoking quantity (number of cigarettes smoked per day (CPD) and pack years), years since quit smoking in former smokers and smoking cessation (former versus current smokers) – among controls of the NHS and HPFS. We performed association analyses separately under an additive model using covariate effects for age, sex and five EVs. The primary gene – smoking status interaction analytic model included SNP, smoking status and SNP × smoking status interaction term, as well as age, sex and the first five principal components as covariates:

*Melanoma diagnosis ~ age + sex +* smoking status + pc.1+ pc.2 +pc.3 + pc.4 + pc.5 + genotype + genotype * smoking status. Both Bonferroni and false discovery rate (FDR) methods were considered for multiple testing issues [40].

## SUPPLEMENTARY MATERIALS



## Abbreviations

SNP: Single nucleotide polymorphism
GWAS: genome-wide association studies
HPFS: Health Professionals Follow-up Study
NHS: Nurses’ Health Study
CHRNA3: cholinergic receptor, nicotinic alpha 3
CHRNA5: cholinergic receptor, nicotinic alpha 5
OR: Odds ratio
SD: Standard deviation.
