# Supplementary material for: Associations between smoking behavior-related alleles and the risk of melanoma
Source: Oncotarget. 2016 Jun 17;7(30):47366–75. doi: 10.18632/oncotarget.10144 (PMC5216947; doi:10.18632/oncotarget.10144)
Supplement: Supplementary file 1 [file oncotarget-07-47366-s001.pdf]

Associations between smoking behavior-related alleles and the risk of melanoma

Supplementary Materials

SUPPLEMENTARY METHODS

Harvard NHS & HPFS set

Description of eight GWAS study populations of the NHS & HPFS set

1) *Postmenopausal invasive breast cancer case-control study nested within the NHS (CGEMS):* Eligible cases in this study consisted of women with pathologically confirmed incident breast cancer from the sub-cohort who gave a blood specimen. Cases with a diagnosis after blood collection up to June 1, 2000 with no previously diagnosed cancer except for non-melanoma skin cancer were included. One control for each case was randomly selected among women who gave a blood sample and were free of diagnosed cancer (excluding non-melanoma skin cancer) up to and including the interval in which the case was diagnosed. Controls were matched to cases on year of birth, menopausal status, recent post-menopausal hormone (PMH) use, month of blood return, time of day of blood collection, and fasting status at blood draw [1].

2) *Type 2 diabetes (T2D) case-control study nested within the NHS and HPFS (T2D_NHS and T2D_HPFS):* Diabetes cases were defined as self-reported incident diabetes confirmed by a validated supplementary questionnaire. For cases before 1998, diagnosis was made using criteria consistent with those proposed by the National Diabetes Data Group (NDDG). For cases during the 1998 and 2000 cycles, the American Diabetes Association's diagnostic criteria were used for the diagnosis of diabetes cases. The non-diabetic control subjects were matched to cases on age, month and year of blood draw, and fasting status [2].

3) *Coronary heart disease (CHD) case-control study nested within the NHS and HPFS (CHD_NHS and CHD_HPFS):* In both the NHS and HPFS, participants who had reported an incident CHD event on the follow-up questionnaire were contacted for confirmation, and permission to review medical records was requested. Medical records for deceased participants were also sought for deaths that were identified by families and postal officials and through the National Death Index. Physicians blinded to the participant's questionnaire reports reviewed all medical records. Fatal CHD cases were identified primarily through review of medical records, as previously described [3]. Among participants who provided blood samples and who were without cardiovascular disease or cancer at blood draw, incident CHD cases occurring after blood draw were selected as cases. Controls were selected, in a 2:1 ratio matched to cases on age, smoking, and month of blood return.

4) *Kidney stone study nested within the NHS and HPFS (KS_NHS_HPFS):* Participants from the KS_NHS_HPFS were individuals who performed a 24-hour urine collection; two-thirds had a history of incident nephrolithiasis. Details regarding the urine collection [4] and the confirmation of kidney stone disease were published previously [5]. The participants reported on the interval diagnosis of kidney stones every 2 years. Any study participant who reported a new kidney stone was sent an additional questionnaire to determine the date of occurrence and the symptoms produced by the stone.

5) *Prostate cancer study nested within the HPFS (ADVCAP_HPFS):* Prostate cancer cases are matched to controls on birth year (+/−1) and ethnicity. Controls are selected from those who are cancer-free at the time of the case's diagnosis, and had a prostate- specific antigen test after the date of blood draw.

6) *Glaucoma:* Primary open angle glaucoma cases were defined as self-reported incident glaucoma confirmed to be primary open angle glaucoma with reproducible visual field loss confirmed with standardized visual field tests and validated supplementary questionnaires or medical records from diagnosing eye care providers. Cases were all Caucasian and matched to controls (roughly 1:1) on cohort, age, type of sample (blood or cheek cell).

7) *PANSCAN:* Subjects were followed prospectively with repeated assessments of lifestyle factors and ascertainment of cancer diagnoses. Each cohort study selected participants with blood or buccal cells collected before cancer diagnosis. One control was selected per case within each cohort. Controls were matched on year of birth (± 5 years), gender, self-reported race/ethnicity, and source of DNA (peripheral blood or buccal cells). Controls were alive without pancreatic cancer on the incidence date of the matched case [6].

Quality control (QC) procedures for eight GWAS of the NHS & HPFS set

1) *CGEMS:* Detailed QC procedures were provided previously [1]. Briefly, a total of 555,352 SNP genotype assays were attempted on the 2,494 DNA samples using the Illumina HumanHap550 chip. Whenever the completion rate for a sample was below 90%, the sample was assayed a second time. Samples that did not meet the 90% completion threshold after a second attempt were excluded from further analysis. We excluded 59 samples from NHS (30 cases and 29 controls) from further analysis based on these criteria, which left 2,435 DNAs for the subsequent analyses.

A total of 8,706 SNPs (~1.57% overall) failed to provide accurate genotype results owing to either a lack of calls or low call rates (< 90%). We performed further quality control analysis on the remaining 546,646 SNPs. The genotyping of the SNPs with high call rate on the 2,412 NHS DNAs with high completion rate generated 1.27 billion genotype calls. For this set of SNPs and samples, the percentage of missing data was < 1%. The genotype concordance rate for SNP assays was evaluated using the 93 pairs of known duplicated DNAs from the NHS. These pairs of DNAs were separate aliquots from the same DNA preparation; all met quality control criteria required for the other DNAs, thereby providing reliable data for comparison. Analysis of the discrepancies within these pairs of DNA uncovered results similar to those of the Centre d'Etude du Polymorphisme Humain (CEPH) DNA duplicates reported in the prostate cancer CGEMS GWAS [7]. We observed an average concordance rate of 99.985% (50,820,003 concordant genotype calls out of 50,827,468 comparisons).

2) *T2D_NHS and T2D_HPFS*: Detailed QC procedures were provided previously [2]. Briefly, the NHS and HPFS T2D GWA scans are a component of the Gene Environment- Association Studies (GENEVA) under the NIH Genes, Environment and Health Initiative (GEI). Genotypic data first passed Broad's initial QC which included SNP fingerprints for sample tracking and early detection of sample misidentification, missing call rates of ≥ 5%, the use of a HapMap control to check genotype quality independent of study samples and tracking of reagent and instrumental performance.

Genotype data were subsequently released for further QC to the GENEVA Coordinating Center at the University of Washington. Relatedness was evaluated using pairwise identity-by descent estimation using 80k SNPs in a method of moments approach implemented in PLINK software [8]. Of the 909,622 SNP probes on the array, 879,071 passed the Broad's technical QC standards for NHS samples, and 874,517 SNP probes passed this QC stage for HPFS samples. We applied the same QC parameters to both scans: excluding SNPs which were monomorphic, had a missing call rate of ≥ 2%, more than one discordance, significant deviations from HWE ($P < 1 \times 10^{-4}$) and an MAF of < 0.02. Duplicate SNPs (assayed with different probes) were also removed. A total of 704,409 SNPs for NHS samples and 706,040 SNPs for HPFS samples passed QC.

3) *CHD_NHS and CHD_HPFS*: QC procedures for CHD_NHS and CHD_HPFS were similar to those used for T2D. Briefly, PLINK software [8] was used for data cleaning. The data were cleaned separately for NHS and HPFS according to thresholds recommended by the GENEVA consortia described above. We excluded SNPs that met any of following criteria: 1) MAF < 0.02; 2) call rate < 95%; 3) P for Hardy-Weinberg equilibrium HWE < 0.00001 in control groups; 4) concordance rate < 95% among the duplicated QC samples; 5) significant difference in missing rates between cases and controls ($P < 0.00001$). After applying the QC filter, 721,316 SNPs remained for NHS and 724,881 for HPFS.

4) *KS*: All samples were highly genotyped. SNPs with MAF < 1.0% in either cases or controls were removed, as were SNPs with less than 97% completion in cases or controls or any SNP with HWE-test $p < 10^{-4}$.

5) *ADVCAP_HPFS*: Samples were excluded if the genotyping call rate was ≤ 95%. Samples with autosomal heterozygosity < 0.25 or > 0.35 were excluded. SNPs with MAF < 2.5% in either cases or controls were removed, as were SNPs with less than 95% completion in cases or controls or any SNP with HWE-test $p < 10^{-5}$. We had 519,982 SNPs after filtering.

6) *GLAUCOMA*: Samples were excluded if the genotyping call rate was ≤ 97%. SNPs with MAF < 1.0% in either cases or controls were removed, as were SNPs with less than 95% completion in cases or controls.

7) *PANSCAN*: Samples were excluded if the genotyping call rate was ≤ 97%, no other filter.

8) *MELANOMA*: SNPs with MAF < 1.0% in either cases or controls were removed, as were SNPs with less than 97% completion in cases or controls or any SNP with HWE- test $p < 10^{-4}$.

REFERENCES

1. Hunter DJ, Kraft P, Jacobs KB, Cox DG, Yeager M, Hankinson SE, Wacholder S, Wang Z, Welch R, Hutchinson A, Wang J, Yu K, Chatterjee N, et al. A genome-wide association study identifies alleles in FGFR2 associated with risk of sporadic postmenopausal breast cancer. Nature genetics. 2007; 39:870–874.
2. Qi L, Cornelis MC, Kraft P, Stanya KJ, Linda Kao WH, Pankow JS, Dupuis J, Florez JC, Fox CS, Pare G, Sun Q, Girman CJ, Laurie CC, et al. Genetic variants at 2q24 are associated with susceptibility to type 2 diabetes. Hum Mol Genet. 2010; 19:2706–2715.

3. Rimm EB, Giovannucci EL, Willett WC, Colditz GA, Ascherio A, Rosner B, Stampfer MJ. Prospective study of alcohol consumption and risk of coronary disease in men. Lancet. 1991; 338:464–468.
4. Curhan GC, Taylor EN. 24-h uric acid excretion and the risk of kidney stones. Kidney international. 2008; 73:489–496.
5. Taylor EN, Stampfer MJ and Curhan GC. Obesity, weight gain, and the risk of kidney stones. Jama. 2005; 293:455–462.
6. Wolpin BM, Kraft P, Gross M, Helzlsouer K, Bueno-de-Mesquita HB, Steplowski E, Stolzenberg-Solomon RZ, Arslan AA, Jacobs EJ, Lacroix A, Petersen G, Zheng W, Albanes D, et al. Pancreatic cancer risk and ABO blood group alleles: results from the pancreatic cancer cohort consortium. Cancer Res. 2010; 70:1015–1023.
7. Yeager M, Orr N, Hayes RB, Jacobs KB, Kraft P, Wacholder S, Minichiello MJ, Fearnhead P, Yu K, Chatterjee N, Wang Z, Welch R, Staats BJ, et al. Genome-wide association study of prostate cancer identifies a second risk locus at 8q24. Nature genetics. 2007; 39:645–649.
8. Purcell S, Neale B, Todd-Brown K, Thomas L, Ferreira MA, Bender D, Maller J, Sklar P, de Bakker PI, Daly MJ, Sham PC. PLINK: a tool set for whole-genome association and population-based linkage analyses. American journal of human genetics. 2007; 81:559–575.

Supplementary Table S1: Characteristics of melanoma cases and controls in the MD Anderson set

	MD Anderson set		
	**All** **N = 2830**	**Cases** **N = 1804**	**Controls** **N = 1026**
Male (n, %)	1673 (59.1)	1060 (58.8)	613 (59.7)
Female (n, %)	1157 (40.9)	744 (41.2)	413 (40.3)
Age (mean ± SD)	51.77 ± 13.92	52.03 ± 14.62	51.31 ± 12.59

Supplementary Table S2: Association between smoking behaviors–related SNPs and melanoma risk in each data set

			NHS and HPFS set					MD Anderson set	
**SNP**	**CHR**	**Gene**	**Risk**	**Ref**	**Allele frequency**	**OR**	***P***	**OR**	***P***
rs215605[a]	7	PDE1C	G	T	0.38	0.96	0.54	1.04	0.50
rs11782673[b]	8	PTK2B	G	A	0.17	1.16	0.09	1.04	0.62
rs6474412[a]	8	SMIM19, CHRNB3	C	T	0.23	1.06	0.43	1.00	0.97
rs13280604[a]	8	CHRNB3	G	A	0.23	1.06	0.44	0.99	0.89
rs7872903[a]	9	FAM163B, DBH	C	T	0.21	1.07	0.41	0.96	0.52
rs1329650[a]	10	HECTD2-AS1	T	G	0.28	0.96	0.54	0.98	0.76
rs1013442[a]	11	BDNF-AS	T	A	0.25	1.00	0.98	1.04	0.59
rs6265[a]	11	BDNF	T	C	0.19	1.05	0.59	1.04	0.58
rs2036534[b]	15	HYKK	C	T	0.22	1.14	0.10	**1.14**	**0.06**
rs588765[b]	15	CHRNA5	T	C	0.42	1.03	0.62	1.02	0.78
rs16969968[a]	15	CHRNA5	A	G	0.35	**0.87**	**0.05**	0.93	0.22
rs578776[a]	15	CHRNA3	A	G	0.28	**1.16**	**0.03**	1.02	0.73
rs1051730[a]	15	CHRNA3	A	G	0.34	0.89	0.11	0.93	0.22
rs6495308[a]	15	CHRNA3	C	T	0.23	1.13	0.12	1.05	0.43
rs7937[a]	19	MIA-RAB4B	C	T	0.44	0.89	0.08	0.97	0.61
rs3733829[a]	19	RAB4B-EGLN2	G	A	0.36	0.99	0.85	0.94	0.26

a. SNPs reached genome-wide significance (P value = 5×10^{-8}) with smoking behaviors.
b. SNPs did not reach P value of 5×10^{-8}.
